# Difficult to treat? A comparison of the effectiveness of treatment as usual in refugees and non-refugees

**DOI:** 10.1192/pb.bp.114.047928

**Published:** 2015-08

**Authors:** F. Jackie June ter Heide, Geert E. Smid

**Affiliations:** 1 Foundation Centrum '45/partner in Arq, The Netherlands

## Abstract

**Aims and method** To examine treatment response in traumatised refugees, we compared routine outcome monitoring data (Harvard Trauma Questionnaire) of two refugee populations with those of individuals experiencing profession-related trauma who were treated at a specialised psychotrauma institute.

**Results** Asylum seekers/temporary refugees (*n* = 21) and resettled refugees (*n* = 169) showed significantly lower post-traumatic stress disorder (PTSD) symptom reduction between intake and 1 year after intake than did a comparison group of non-refugees (*n* = 37), but the interaction effect was clinically small (partial η^2^ = 0.03). Refugees who had more severe symptoms at intake showed significantly greater symptom reduction after 1 year.

**Clinical implications** Therapists and refugee patients should have realistic expectations about response to treatment as usual. Additional treatment focusing on improving quality of life may be needed for refugees whose PTSD symptom severity remains high. At the same time, novel approaches may be developed to boost treatment response in refugee patients with low responsiveness.

Among many clinicians, traumatised asylum seekers and refugees have a reputation of being difficult to treat. Low treatment response in refugees is often attributed to patient-related factors,^1^ such as trauma history, current stressors and complex psychopathology. Many asylum seekers and refugees have been exposed to multiple, prolonged, interpersonal traumatic events such as war and human trafficking.^2^ In addition, they have to handle the stress of forced migration, including involvement in legal procedures^3^ and loss of their home country, cultural resources, family and social status.^4^ Apart from post-traumatic stress disorder (PTSD),^5^ they may experience comorbid symptoms including depression, anxiety and psychosis,^6^ as well as symptoms sometimes referred to as complex PTSD.^7^ By contrast, some clinicians argue that it is the treatment offered to refugees, rather than their potential to benefit from treatment, that leads to low treatment response,^8^ and that refugees, like other adults with chronic PTSD,^9^ should be treated with trauma-focused interventions. One way to examine treatment response in refugees is by comparing the effectiveness of different kinds of treatment in refugee samples. In recent years, randomised trials have shown promising effects for trauma-focused treatment in refugees.^10^ Another way to examine treatment response is to compare the effectiveness of treatment in refugee samples and non-refugee samples. This has been done little, if at all. This study's aim is to compare traumatised asylum seekers' and refugees' response to treatment as usual with that of another multiply traumatised population: indivduals affected by profession-related trauma (i.e. military veterans and police officers).

## Method

### Setting

Data were collected in Foundation Centrum '45, a Dutch mental health institute specialising in treatment of complex psychotrauma. Specific populations include asylum seekers and refugees, veterans of various peace missions, World War II resistance fighters as well as concentration camp survivors and their offspring, and police officers. Centrum '45 receives national referrals of patients who, owing to their psychosocial complexity, cannot be treated in general mental healthcare or who have shown insufficient response to treatment in general mental healthcare. Treatment for PTSD (individually or in groups) generally consists of a combination of supportive therapy, pharmacotherapy and trauma-focused therapy, particularly eye movement desensitisation and reprocessing (EMDR),^11^ narrative exposure therapy (NET)^12^ and brief eclectic psychotherapy for PTSD (BEPP).^13^ As these three trauma-focused treatments are evidence based, choice of treatment mainly depends on the therapist's training. Art therapy, psychomotor therapy and music therapy are also offered, especially to patients who follow a clinical or day-clinical programme.

### Assessments

To evaluate the effectiveness of treatment as usual, Centrum '45 has routinely administered assessments at intake and at the end of treatment. Since 2007, a routine outcome monitoring (ROM) assessment 1 year after intake has been added for all patient populations. Since its introduction, ROM response has increased from around 40 to 55% in 2012. For several years, the Harvard Trauma Questionnaire (HTQ)^14^ was used as a ROM instrument with refugees and for a shorter period also with non-refugee populations. The HTQ has been specifically designed for use with refugee populations. It is a self-report instrument that consists of two parts: one focusing on traumatic events and one on symptoms of post-traumatic stress (specific to DSM-IV^15^ and additional symptoms reported by traumatised refugees). Symptoms are rated on a four-point scale ranging from 1 (not at all) to 4 (extremely). A mean score of 2.5 has been recommended as cut-off score for PTSD,^16^ although this recommendation has not been validated in a wide range of patient populations.

### Sample

To answer our research question, we had at our disposal a ROM data-set that consisted of 577 patients who had completed assessments both at intake and 1 year after intake (with a range of 8 to 16 months). From this data-set, we excluded all partners and children of war-affected persons (*n* = 218; primarily children of parents traumatised in World War II) because their reasons for seeking help, generally speaking, do not include PTSD. We then excluded all patients who at intake had not been administered the HTQ (*n* = 125) but another instrument to assess PTSD (a Dutch self-rating inventory for PTSD).^16^ As the final dataset contained only a small number of patients traumatised during World War II (*n* = 7), we also excluded those patients. The final data-set consisted of 227 patients who had had their second assessment between March 2007 and April 2013. We divided the sample into three groups: asylum seekers/temporary refugees (i.e. those who are still awaiting the decision on their asylum application and those who have obtained temporary refugee status, which may not be extended after 5 years), resettled refugees (i.e. those who have obtained permanent refugee status or subsequent Dutch nationality), and patients with profession-related trauma (i.e. military veterans and police officers).

### Statistical analysis

All analyses were performed using SPSS version 20.0 for Windows. Demographical and clinical characteristics were calculated, and chi-squared and *t*-tests were conducted to check for demographical and clinical differences between the groups. For the HTQ, mean PTSD severity at intake (T1) and one year after intake (T2) was computed as well as the difference between the two (PTSD symptom reduction). We checked HTQ variables for extreme outliers, but we found none. Missing data for the HTQ consisted of missing mean scores at T2 for 7 patients (2 asylum seekers/temporary refugees and 5 resettled refugees) and missing events scores at T1 for 42 patients (4 asylum seekers/temporary refugees, 34 resettled refugees and 4 professionals). We handled missing data by using pair-wise deletion.

We conducted pair-wise *t*-tests to determine treatment response within each group, and calculated by hand the effect sizes (η^2^). Following Cohen, we interpreted η^2^ = 0.01 to be a small effect, η^2^ = 0.06 as moderate and η^2^ = 0.14 as large.^17^ We set confidence intervals at 95%. To examine potential differences in treatment response between the three groups, we conducted repeated measures analysis of variance (ANOVA), using time as within-subjects factor and group as between-subjects factor. For the interaction effect, an effect-size (partial η^2^) of 0.01 was interpreted to be small, 0.09 as medium and 0.25 as large.^17^ To examine variables associated with treatment response in asylum seekers and refugees, we performed a multiple regression analysis with PTSD symptom reduction (HTQ score at T1 minus HTQ score at T2) as the dependent variable and demographic variables (gender, age and refugee status (no/temporary/permanent)) and clinical variables (PTSD severity at T1, number of traumatic event types and time between assessments) as independent variables.

## Results

### Demographic characteristics

For demographic and clinical characteristics of the final sample, see Table 1.

**Table 1:** Demographic and clinical characteristics of the groups

	Asylum seekers/ temporary refugees (*n* = 21)	Resettled refugees (*n* = 169)	Profession- related trauma (*n* = 37)	*F*	d.f.	*P*
Demographic characteristics						
Age, years: mean (s.d.)	36.1 (10.4)	43.8 (8.9)	44.5 (8.6)	7.32	2	0.001
Male, *n* (%)	12 (57.1)	123 (72.8)	34 (91.9)		2	0.009^a^

Clinical characteristics						
HTQ score at intake, mean (s.d.)	3.14 (0.35)	3.08 (0.52)	2.80 (0.53)	6.72	2	0.002
Traumatic event types (HTQ),^b^ *n*: mean (s.d.)	13.9 (4.0)	12.3 (5.4)	9.3 (4.6)	8.12	2	0.001
Time between assessments, months: mean (s.d.)	12.1 (1.7)	12.1 (1.4)	10.6 (1.5)	16.09	2	<0.001

HTQ, Harvard Trauma Questionnaire.

a. χ^2^ = 9.47.

b. Experienced or witnessed.

Because this study was observational, we found significant differences in demographic and clinical characteristics between the three groups for all variables. Asylum seekers/temporary refugees came predominantly from Afghanistan, Armenia, Iraq and Sierra Leone (*n* = 3, 14.3% for each country); resettled refugees came predominantly from the former Yugoslavia (*n* = 59, 34.9%), Iraq (*n* = 28, 16.6%) and Afghanistan (*n* = 22, 13.0%); and patients who had profession-related trauma came predominantly from The Netherlands (*n* = 33, 89.2%). The traumatic events that the asylum seekers/temporary refugees most frequently reported were physical torture (*n* = 17, 81.0%), threat of physical torture (*n* = 17, 81.0%) and other life-threatening situation (*n* = 17, 81.0%). Resettled refugees most frequently reported being close to death (*n* = 135, 79.9%), other life-threatening situation (*n* = 125, 74.0%) and forced isolation from family (*n* = 123, 72.8%). Professionals most frequently reported other life-threatening situation (*n* = 34, 91.9%), combat situation (*n* = 34, 91.9%), serious injury (*n* = 31, 83.8%) and being close to death (*n* = 31, 83.8%).

### Treatment outcome

**Fig. 1:**
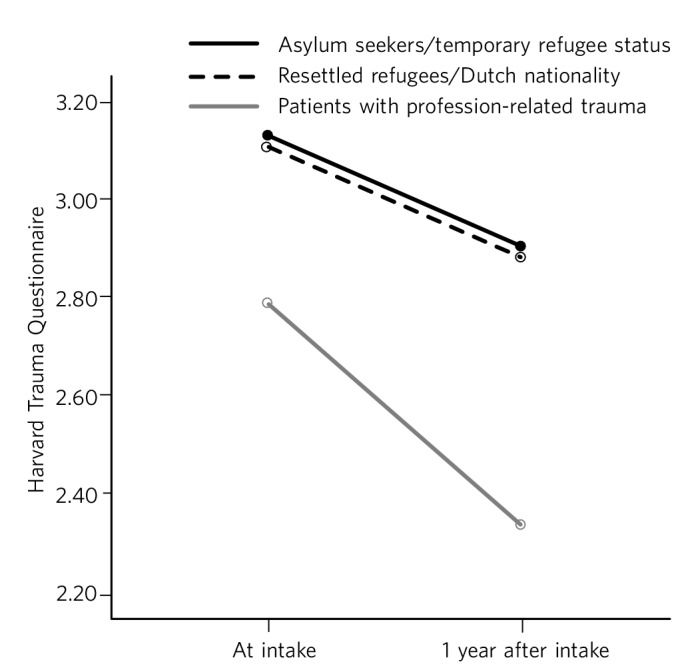
PTSD symptom severity at intake and after 1 year.

Figure 1 shows the results of the repeated measures ANOVA for the three groups.

Mean PTSD symptom severity decreased from 3.13 (95% CI 2.91 to 3.35) to 2.92 (95% CI 2.65 to 3.20) for asylum seekers/temporary refugees; from 3.10 (95% CI 3.03 to 3.18) to 2.88 (95% CI 2.79 to 2.98) for resettled refugees; and from 2.80 (95% CI 2.64 to 2.96) to 2.31 (95% CI 2.11 to 2.51) for patients suffering from profession-related trauma. Paired-samples *t*-tests revealed a significant decrease in PTSD severity for resettled refugees (0.22, s.d. = 0.52, *t*_(163)_ = 5.39, *P*<0.001) and for professionals (0.49, s.d. = 0.64, *t*_(36)_ = 4.65, *P*<0.001), but not for the smallest group, asylum seekers/temporary refugees (0.21, s.d. = 0.59, *t*_(18)_ = 1.53, *P* = 0.143). Effect sizes for treatment response in asylum seekers/temporary refugees and resettled refugees were moderate (η^2^ = 0.12 and η^2^ = 0.15, respectively); effect size for patients with profession-related trauma was large (η^2^ = 0.38).^16^ Repeated measures ANOVA showed a significant effect for time (*F* = 32.27, *P*<0.001) with a medium effect size (partial η^2^ = 0.13), and a significant group×time interaction effect (*F* = 3.65, *P* = 0.028) with a small effect size (partial η^2^ = 0.03).^16^

We then combined the two refugee groups and, using multiple regression analysis, we examined whether seven demographic and clinical variables were associated with PTSD symptom reduction (Table 2).

**Table 2:** Factors associated with reduction in PTSD symptom severity in refugees after 1 year

	B	95% CI	β	*P*
Demographic variables				
Gender	0.11	−0.07 to 0.29	0.09	0.238
Age	0.00	−0.01 to 0.00	−0.09	0.277

Refugee status				
None *v.* permanent	−0.03	−0.37 to 0.31	−0.01	0.872
Temporary *v.* permanent	−0.14	−0.52 to 0.24	−0.06	0.464

Clinical variables				
PTSD symptom severity at intake (HTQ)	0.48	0.32 to 0.64	0.45	<0.001
Traumatic event types (HTQ), *n*	−0.01	−0.03 to 0.00	−0.11	0.157
Time between assessments, months	−0.01	−0.06 to 0.05	−0.01	0.851

B, regression coefficient; β, standardised regression coefficient; HTQ, Harvard Trauma Questionnaire; PTSD, post-traumatic stress disorder

As shown in Table 2, refugee patients with more severe PTSD symptoms at intake had significantly stronger reductions in PTSD symptom severity after 1 year. The other variables were not significantly associated with PTSD symptom reduction. The percentage of variance explained by the model (R^2^) was 21.5%.

## Discussion

This study shows that asylum seekers/temporary refugees and resettled refugees experienced significantly lower PTSD symptom reduction between intake and 1 year after intake than did a comparison group of multiply traumatised military veterans and police officers. However, greatest differences between groups were found in PTSD symptom severity at intake and 1 year after intake rather than in PTSD symptom reduction. Explorations of PTSD symptom reduction in refugees showed that those who had more severe symptoms at intake experienced significantly greater symptom reduction after 1 year; other variables (including variables related to refugee status and number of traumatic events) were not related to symptom reduction.

The results show that despite specialised treatment being offered to refugees, treatment response can be limited and PTSD severity frequently remains high. Possible explanations, and consequently clinical implications, might be threefold: patient-related, therapist-related and treatment-related. As for patient-related factors, the multiple determinants of PTSD might influence refugees' ability to benefit from treatment. It is generally acknowledged that PTSD in refugees is influenced by both traumatic and current stressors, some (or many) of which may be beyond the patients' and therapists' control.^18^ Following this explanation, clinicians and patients should have realistic expectations about what treatment may achieve in such a heavily traumatised and burdened population. Interventions that focus on improving quality of life rather than on further symptom reduction, such as acceptance and commitment therapy,^19^ might be useful for those patients who despite prolonged treatment continue to suffer from clinically significant PTSD. Clinicians sometimes suspect asylum seekers to exaggerate symptoms to remain in medical care and thereby increase the chance of obtaining a refugee status. We found no substantiation for this hypothesis of ‘secondary gain’ - in our study, not having a permanent refugee status was not associated with a decreased treatment response.

As for therapist-related factors, therapeutic skills that might suffice in trauma-focused treatment of other multiply traumatised groups might fall short in the treatment of refugees. Therapists might need more extensive training and supervision regarding choosing and staying with a treatment focus, categorising and selecting of target memories, and understanding and restructuring of trauma-related cognitions in order not to lose their way in the multitude of symptoms, memories and transcultural challenges. At the same time, therapists need to maintain a sense of being ‘good enough’ to provide treatment to refugees with limited responsiveness.^20^

Finally, regarding treatment-related factors, not all evidence-based treatments will work with all refugees. Therapists will need to explore non-response, and they may need to consult refugee patient populations themselves^21^ to examine which treatment aims and techniques speak to refugees who insufficiently benefit from treatment as usual. In addition, novel approaches may be developed to enhance treatment response. Centrum '45 is currently exploring the feasibility of refugee treatment that focuses primarily on prolonged grief rather than on PTSD, and of intranasal oxytocin as a novel strategy to boost treatment response in refugees.^22^

### Limitations

Although this study is valuable for comparing the effects of treatment as usual in refugee populations with those in a non-refugee population (which, to our knowledge, has not been done before), it also has several limitations. First, a division of the asylum seeker group into asylum seekers and temporary refugees, and of the profession-related trauma group into military veterans and police officers, would have been clinically meaningful but was not possible due to limited sample sizes for these groups. Second, some variables that might have shed light on differences in treatment response between the three groups (including comorbid disorders, the amount and content of treatment, change in refugee status and chronicity of PTSD) were not included in the data-set. Future studies should use a broader range of variables to more comprehensively assess predictors of refugees' treatment responses. Third, ROM assessments at our institute are completed by about 55% of patients, and findings might not generalise to our complete patient population, nor to traumatised refugees in general.

Nevertheless, our study contributes to the debate on refugees' treatment response by showing that it is indeed relatively lower than that of multiply traumatised non-refugees.
